# cAMP/Protein Kinase A Activates Cystic Fibrosis Transmembrane Conductance Regulator for ATP Release from Rat Skeletal Muscle during Low pH or Contractions

**DOI:** 10.1371/journal.pone.0050157

**Published:** 2012-11-30

**Authors:** Jie Tu, Lin Lu, Weisong Cai, Heather J. Ballard

**Affiliations:** 1 Department of Physiology, The University of Hong Kong, Pokfulam, Hong Kong, Special Administrative Region, People's Republic of China; 2 Shenzhen Key Laboratory for Neuropsychiatric Modulation, Shenzhen Institutes of Advanced Technology, Chinese Academy of Sciences, Shenzhen, Guangdong, People's Republic of China; 3 Institute of Cardiovascular Science and Medicine, The University of Hong Kong, Pokfulam, Hong Kong, Special Administrative Region, People's Republic of China; University of Pittsburgh, United States of America

## Abstract

We have shown that cystic fibrosis transmembrane conductance regulator (CFTR) is involved in ATP release from skeletal muscle at low pH. These experiments investigate the signal transduction mechanism linking pH depression to CFTR activation and ATP release, and evaluate whether CFTR is involved in ATP release from contracting muscle. Lactic acid treatment elevated interstitial ATP of buffer-perfused muscle and extracellular ATP of L6 myocytes: this ATP release was abolished by the non-specific CFTR inhibitor, glibenclamide, or the specific CFTR inhibitor, CFTR_inh_-172, suggesting that CFTR was involved, and by inhibition of lactic acid entry to cells, indicating that intracellular pH depression was required. Muscle contractions significantly elevated interstitial ATP, but CFTR_inh_-172 abolished the increase. The cAMP/PKA pathway was involved in the signal transduction pathway for CFTR-regulated ATP release from muscle: forskolin increased CFTR phosphorylation and stimulated ATP release from muscle or myocytes; lactic acid increased intracellular cAMP, pCREB and PKA activity, whereas IBMX enhanced ATP release from myocytes. Inhibition of PKA with KT5720 abolished lactic-acid- or contraction-induced ATP release from muscle. Inhibition of either the Na^+^/H^+^-exchanger (NHE) with amiloride or the Na^+^/Ca^2+^-exchanger (NCX) with SN6 or KB-R7943 abolished lactic-acid- or contraction-induced release of ATP from muscle, suggesting that these exchange proteins may be involved in the activation of CFTR. Our data suggest that CFTR-regulated release contributes to ATP release from contracting muscle in vivo, and that cAMP and PKA are involved in the activation of CFTR during muscle contractions or acidosis; NHE and NCX may be involved in the signal transduction pathway.

## Introduction

Adenosine was first proposed as a mediator of exercise hyperaemia more than 50 years ago [1]. Interstitial adenosine is increased during contractions of both red and white muscles [2]–[4], which is estimated to account for about 40% of the vasodilation [5]–[8]. Interstitial adenosine is formed extracellularly [9], [10]: an increase in the interstitial adenine nucleotides is the main driving force for the increased adenosine formation during muscle contractions [9]. Several authors have documented the increase in interstitial ATP during muscle contractions [2], [11], [12], but the mechanism by which ATP is released from contracting skeletal muscle cells is unknown.

ATP itself is also considered to be an important extracellular signalling molecule [13]: as well as giving rise to vasodilator quantities of adenosine, ATP can induce vasodilation directly through its action on endothelial P2Y receptors and by inhibition of sympathetic vasoconstriction [14], [15], and it can activate the muscle pressor reflex through its action on P2X receptors [16].

We previously reported that lactic acid stimulated ATP release from skeletal muscle through a mechanism that involved the cystic fibrosis transmembrane conductance regulator (CFTR) [17], and that muscle pH was negatively correlated with the extracellular adenosine or ATP concentrations [17]–[19]. We proposed that the decrease in pH during muscle contractions stimulated ATP release from muscle through a CFTR-dependent mechanism, with that ATP then being converted to adenosine in the interstitial space to bring about the muscle vasodilation [17].

CFTR is a member of the ATP-binding cassette (ABC) superfamily of proteins as well as a chloride channel (for recent review see [20]). CFTR is unique among the ABC transporters because it exhibits ligand gating, conferred by the presence of a central ∼200-residue regulatory (R) domain containing multiple serines that can be phosphorylated by cAMP-dependent protein kinase A (PKA), which facilitates its ATP binding; ATP hydrolysis then triggers the opening and closing of the Cl^−^ channel [20]. The regulation of skeletal muscle CFTR activity has not been investigated previously.

Many studies have reported the involvement of ABC proteins in ATP release [21]–[23]: CFTR-regulated ATP release has been observed in a variety of native cell types, including erythrocytes and epithelial cells [24], [25], whilst transfection of carcinoma cells with CFTR or reconstitution of CFTR into lipid bilayers is associated with the appearance cAMP-dependent ATP release [26]–[28]. The mechanism by which CFTR facilitates the ATP release from muscle remains controversial: some authors have proposed that ATP leaves the cell through CFTR itself, whilst others propose that CFTR regulates the activity of a separate ATP channel protein [23], [29], [30].

Here, we investigate the signal transduction mechanism linking the decrease in pH to CFTR activation, and assess whether CFTR is involved in ATP release from muscle during contractions in-vivo. We hypothesised that increased activity of the Na^+^/H^+^ exchanger (NHE) at low pH increased intracellular sodium, which in turn led to increased activity of the Na^+^/Ca^2+^ exchanger (NCX), producing an area of elevated intracellular calcium close to the cell membrane. According to our model, this calcium could then activate adenyl cyclase (AC), increasing cAMP, and activate CFTR through the PKA pathway. This model has been tested using inhibitors of the proposed signal transduction pathway, and our findings are consistent with the suggested model.

The involvement of CFTR in ATP release from contracting muscle was investigated using microdialysis to sample the interstitial fluid of blood-perfused gastrocnemius muscles of anaesthetised rats at rest and during muscle contractions: the increase in interstitial ATP during muscle contractions was compared in the absence or presence of inhibitors of CFTR itself, or of key elements in its proposed activation pathway. The findings are consistent with the proposal that CFTR regulates ATP release from muscle during contractions.

## Materials and Methods

### Materials

Chemicals were purchased from Sigma, USA. Stock standard solutions of ATP, ADP, AMP and adenosine were dissolved in ultrapure water. Stock solution of forskolin was prepared in phosphate-buffered saline (PBS). Stock solutions of the other pharmacological agents (CFTR_inh_-172, SN6, KB-R7943, amiloride and KT5720) were prepared in DMSO. The final DMSO concentration did not exceed 1 µl per ml of the perfusion buffer or bathing medium.

### Culture of rat L6 skeletal myoblasts

L6 cells obtained from ATCC (Manassas, VA, USA) were cultured and maintained in a complete Dulbecco's Modified Eagle Medium (DMEM) containing 10% heat-inactivated fetal bovine serum (FBS), and 1% penicillin/streptomycin (all from Gibco) at 37°C in a humidified 5% CO_2_ in air atmosphere. Prior to beginning the experimental procedures, the myoblasts were seeded into 6-well plates in 1 ml of culture medium containing serum and antibiotics at a density of 3×10^5^ cells per well, and a further 2 ml of complete growth medium was added. Cells were incubated at 37°C in a humidified 5% CO_2_ in air atmosphere for 24 hours prior to protocols leading to the analysis of intracellular metabolites, or for 48 hours (which allowed them to grow to 80–90% confluence) prior to measurements of extracellular ATP; the cells were then washed with PBS, 1 ml of fresh medium without serum was added to each well, and the experimental treatment was begun.

### Ethics statement

All animal protocols were approved by the University of Hong Kong Committee on the Use of Live Animals in Teaching and Research, and all experimental procedures involving animals were carried out in strict accordance with the animal use guidelines of the above committee. Experiments were performed under full anaesthesia, and every effort was made to minimise animal suffering.

### Animal models

Male Sprague-Dawley rats (300 to 450 g) were anaesthetised with pentobarbital sodium (60–70 mg/kg i.p., Sagatal, RMB, Animal Health Ltd, Dagenham, UK). A Y-shaped cannula was placed in the trachea to maintain the airways patent, and animals were allowed to breathe spontaneously. An external jugular vein was cannulated for the administration of supplementary doses of pentobarbital sodium (12 mg/kg whenever toe pinch elicited a withdrawal reflex) and other drugs. Body temperature was maintained between 37.5 and 38.5°C by a heating pad and an external heat lamp.

#### Constant-flow buffer-perfused hindlimb model

The surgical preparation was carried out as previously described [17]. The hindquarters were perfused at 1.5 ml/min via the abdominal aorta with modified Krebs Henseleit buffer (pH 7.4) equilibrated against 95% O_2_/5% CO_2_. Venous drainage occurred through a cannula in the inferior vena cava: samples of venous effluent were collected at intervals for venous pH measurement. Skin was removed from a hindlimb to expose the calf muscles, and a microdialysis probe (LM10, Bioanalytical Systems, West Lafayette Inc, USA) was inserted longitudinally into the soleus muscle. The exposed muscle was wrapped in the plastic film to avoid dehydration.

The microdialysis probe was perfused at 2 µl/min with a fluid of similar composition to the interstitial fluid. Both the hindlimb muscle and the microdialysis probe was perfused for 90 minutes before the experimental procedures and sample collection were begun. The dialysate was collected every 10 minutes in an ice-cooled vial for HPLC analysis of ATP.

#### Free-flow blood-perfused hindlimb model

The blood vessels were left intact, and natural blood perfusion was permitted. The skin was opened down the posterior aspect of one hindlimb, and an LM10 microdialysis probe was inserted longitudinally into the gastrocnemius muscle. The proximal part of the hindlimb was immobilised using two pins, and the foot was fixed securely to an upright post. The distal tendon of the gastrocnemius muscle was tied, cut, and attached to a force transducer (model 50-7915, Harvard, Edenbridge, UK) for recording of the isometric contractile force: signals were amplified using a Harvard transducer amplifier and written onto a Lectromed MX216 recorder (Lectromed, Jersey, UK). An electrode was placed on the sciatic nerve and connected to the stimulator (model S48, Grass Instruments, West Warwick, USA). After the surgical preparation was completed, the skin was sutured back into position to prevent dehydration.

The microdialysis probe was perfused at 4 µl/min with a fluid of similar composition to the interstitial fluid. The microdialysis probe was perfused for 90 minutes before the experimental procedures and sample collection were begun. The dialysate was collected every 10 minutes in an ice-cooled vial for analysis of ATP using a bioluminescent assay.

Microdialysis probe recovery was determined in control experiments from the regression line of dialysate concentration versus probe concentration as previously described [11]. ATP recovery was not altered by the blood flow changes associated with muscle contraction: recovery was 57.7% under resting conditions and 57.1% during contractions. Concentrations shown in this paper have not been corrected for probe recovery.

### Analytical procedures

#### ATP analysis using HPLC

Dialysis samples from the buffer-perfused muscle (15–30 µl) were mixed with 105–90 µl ultrapure water. ATP in the samples was analyzed by ion-pair reverse-phase HPLC as previously described [11]. The ATP peak was identified by its retention time and by comparison of its absorption spectrum to that of the standard. ATP concentrations were quantified using the area under the peak, and corrected for the dilution factor.

#### ATP analysis using a bioluminescence assay

The ATP content of the undiluted samples was determined with the luciferin-luciferase technique using an ATP bioluminescent assay kit (Sigma) in a microplate reader (Infinite M200, Techan, Austria). Samples of the bathing medium from the cultured cells (100 µl) or of the dialysate from the blood-perfused muscle (30 µl) were pipetted into a 96-well microplate. ATP assay solution (100 µl) was added to each well and the luminescent signal was measured within 1 minute. Sample readings were compared to the standard curve for 8×10^−11^–5×10^−8^ M ATP. The standard curve was not significantly altered by any of the inhibitors used in the study. Intracellular ATP was similarly determined using the Sigma Bioluminescent Somatic Cell Assay kit, immediately following the addition of 0.05 ml cell sample to 0.1 ml Somatic Cell ATP Releasing Reagent plus 0.05 ml ultrapure water and mixing [31]. The number of viable cells was determined by Trypan blue exclusion, to permit the determination of per cell ATP content.

#### Western blot

The incubation medium was removed and the L6 cells were washed twice with PBS then 1 ml lysis buffer (150 mM NaCl; 50 mM Tris-HCl; 16 mM NP-40 detergent; 6 mM C_24_H_39_NaO_4_; 3.5 mM SDS; 1 mM Na_3_VO_4_; 1 mM NaF; 1 mM EDTA; 1 mM phenylmethylsulfonyl fluoride (PMSF); 2.3 µM leupeptin; 1.5 µM pepstatin A; 150 nM aprotinin, pH 7.4) was added. The extracted proteins were transferred to a new tube, put on ice for 30 min, then centrifuged at 12,000 rpm for 20 min at 4°C. The protein concentration of the supernatant was assayed using the Bradford assay (Bio-Rad). Equal amounts of protein samples were electrophoresed on 4% stacking gel and 10% separating gel, then transferred to polyvinylidene fluoride (PVDF) membrane (Millipore, Bedford, MA) for blotting by standard protocols using rabbit anti-human polyclonal antibody to residues 55–63 of CFTR (Cell Signaling Technology, Inc., Danvers, MA, USA). The PVDF membrane with protein was then detected using chemiluminescence technology (ECL Plus Western Blotting Reagents, GE Healthcare).

#### RT-PCR

The incubation medium was removed and the L6 cells were washed twice with PBS. Total RNA was extracted using an RNeasy Total RNA Extraction Kit (Qiagen GmbH, Hilden, Germany). cDNA was synthesised from 1 µg of total RNA using an oligo (dT)_18_ primer and M-MuLV Reverse Transcriptase (Fermentas, Canada). cDNA was then amplified using TaqHS DNA polymerase (Takara Bio Inc., Japan); the specific primers and the PCR conditions are listed in Table 1 for CFTR and in Table 2 for AC. After 38 cycles of PCR amplification, the reaction products were separated on a 2% agarose gel and stained with ethidium bromide. The band density was assessed and normalised to that of β-actin (for CFTR) or GADPH (for AC) using Image J software (version 1.43; NIH).

**Table 1:** Specific primers and PCR conditions used for RT-PCR analysis of CFTR mRNA expression.

Gene	GenBank Accession No.	Primer Sequence (5′-3′)	Annealing Temperature (°C)
CFTR (236 bp)	NC_005103	Sense: GTGGCTCCCATTGTCGTAGTAntisense: GGCCGGTTTGTTCTTTATCA	64.0
β-actin (240 bp)	NM_001101	Sense: CACGATGGAGGGGCCGGACTCATCAntisense: TAAAGACCTCTATGCCAACACAGT	60.4

**Table 2:** Specific primers and PCR conditions used for RT-PCR analysis of AC isoform mRNA expression.

AC gene	GenBank Accession No.	Primer Sequence (5′-3′)	Annealing temperature (°C)
mAC 1 (521 bp)	AF053980	sense: CCACGTCCTACATCCTCGTTantisense: AAGTGGTAGGGGCACCTTCT	53
rAC 2 (302 bp)	M80550	sense: CGTGTCACTCTCantisense: CCTTGTTCACATCTGACTC	64
rAC 3 (442 bp)	M55075	sense: CATCGAGTGTCTACGCTTCantisense: GGATGACCTGTGTCTCTTCT	64
rAC 4 (455 bp)	M80633	sense: GGAAGACGAGAAGGGCACCGAGAGantisense: GAGCTGGGGGCCTGGTTGTCAC	64
rAC 5 (498 bp)	M96159	sense: ACCATTGTGCCCCACTCCCTGTTantisense: TCGTCGCCCAGGCTGTAGTTGAA	64
rAC 6 (417 bp)	LO1115	sense: CAAAGGAAGGGACGCCGAGAGGantisense: TGGGGACAGATCACGGGACTAGGA	51
mAC 7 (560 bp)	U12919	sense: CCAGTTATTTAGAGAGAGACCTGantisense: CTTGCTCATCAGGGCCATGCTAA	58
mAC 8 (628 bp)	L26986	sense: TTCACTTGAGCCTAGCCTCGantisense: GGATGTAGATGCGGTGGAAC	55
mAC 9 (312 bp)	U30602	sense: AGCTTATCCTCACCTTCTTCTTCCTCantisense: AGGACACGGTAGCACTCCTTGCC	54

#### CFTR phosphorylation assay

CFTR phosphorylation was measured as described by Chang et al [32]. Cells were washed twice with phosphate-free MEM containing 8% FBS and 50 µM Na_3_VO_4_ and labelled by incubation in 1 ml of the same medium containing 100 µCi [γ-^32^P]-ATP (Perkin-Elmer) for 4 hours. Either forskolin (10 µM) alone or forskolin (10 µM) with dibutyryl-cAMP (200 µM) and IBMX (1 mM) or DMSO vehicle was added to the medium for the last 15 mins of the incubation. Cells were washed twice with PBS, lysed with 1 ml lysis buffer, and CFTR was immunoprecipitated using the M3A7 monoclonal antibody (R & D Systems). Protein G dynabeads (Immunoprecipitation kit, Invitrogen) were incubated with the antibody at 4°C for 1 hour with rotation, then the cell lysate was added and incubation was continued for a further 1 hour, before the magnetic beads were separated using a magnetic particle concentrator (MPC-S, Dynal A.S., Oslo, Norway) and washed. The antibody-protein complex was solubilised in 30 µl buffer by heating at 95°C for 8 min prior to SDS-polyacrylamide gel electrophoresis. The gel was then dried and exposed to x-ray film for 16 hours at −80°C, after which, the film was analysed by densitometry.

#### Phosphor-cAMP response element-binding protein (pCREB) assay

The incubation medium was removed and the cells were washed twice with PBS then lysed. Western blotting was performed as described above. Levels of phospho-CREB (Ser133) and total CREB protein were determined using rabbit anti-phospho-CREB and rabbit anti-CREB antibodies (Cell Signaling Technology, USA) followed by HRP-conjugated goat anti-rabbit IgG (Sigma).

#### Enzyme immunoassay (EIA) for cytoplasmic cAMP measurement

The incubation medium was removed and the L6 cells were washed twice with PBS then lysed. The cAMP concentration in the cell lysate was determined using the non-radioactive cAMP-enzyme immunoassay (EIA) kit from Arbor Assays (Ann Arbor, Michigan, USA) according to the manufacturers instructions. All the experiments were carried out with the acetylated protocol to achieve the maximal sensitivity. The yellow-coloured product formed with the EIA is inversely proportional to the amount of cAMP present in the sample, and was detected at 450 nm using a microplate reader (Synergy™ 4, BioTek Instruments Inc., USA).

#### Protein kinase A (PKA) phosphorylation assay

The incubation medium was removed and the L6 cells were washed twice with PBS then lysed. Lysates were centrifuged at 14,000 g for 5 mins at 4°C. The activity of cAMP-dependent PKA in the supernatant was determined using the phosphorylation assay in the PepTag nonradioactive PKA assay kit (Promega, WI, USA), according to the manufacturer's instructions. Samples were loaded onto 0.8% agarose gels and electrophoresed at 110 V for 20 min.

### Experimental procedures

#### Involvement of CFTR in lactic-acid-induced ATP release from buffer-perfused rat soleus muscle in-vivo

After all surgical procedures had been completed, the muscles were perfused with pH 7.4 buffer for a stabilisation period of at least 90 mins before the sample collection was begun. Two control 10 min samples of microdialysate were first collected. The muscle was then perfused for 20 mins at a time with buffer containing increasing doses (2.5–10 mM) of lactic acid, and two microdialysate samples were collected at each dose. Perfusion with pH 7.4 buffer was then restored for the collection of the two recovery samples. Infusion of one of the blocker substances was begun, and the entire protocol was repeated in the presence of the blocker. The blockers tested were α-cyano-4-hydroxycinnamic acid (2 mM), an inhibitor of the monocarboxylate transporter, CFTR_inh_-172 (20 µM), a specific inhibitor of CFTR, and glibenclamide (200 µM), a non-specific open-pore blocker for CFTR. In a separate series of experiments, the effects of 10 mM lactic acid and 10 mM sodium lactate on interstitial ATP were compared: the muscle was perfused for 20 mins each time with pH 7.4 buffer, 10 mM lactic acid in buffer, pH 7.4 buffer (recovery 1), 10 mM sodium lactate in buffer, and pH 7.4 buffer (recovery 2). Two 10 min microdialysis samples were collected during each treatment. The ATP content of microdialysis samples was analysed by HPLC.

#### Involvement of CFTR in lactic-acid-induced ATP release from cultured L6 myocytes

Cells were seeded into 6-well plates and pre-incubated for 48 hrs. Then the medium was replaced with a serum-free medium. Experimental treatments (lactic acid and/or inhibitors) were added as appropriate, and the plates were returned to the incubator for the duration of the experimental treatment.

In the first series of experiments, the time course of the appearance of ATP in the extracellular medium was examined: 10 mM lactic acid was added to half of the wells on each plate, and the plates were incubated for times of 30 min, 1 hr or 3 hrs before the bathing medium was collected for ATP analysis.

In the second series of experiments, the effect of CFTR inhibitors on the lactic-acid induced increase in extracellular ATP was examined: 10 mM lactic acid was again added to half of the wells on each plate: some plates were left without inhibitors, and either CFTR_inh_-172 (20 µM) or glibenclamide (200 µM) was added to the remaining plates; all plates were incubated for 3 hrs, then the bathing medium was collected for ATP analysis.

In the third series of experiments, the effect of lactic acid on the intracellular ATP was determined: various doses of lactic acid (0–10 mM) were added to the wells, and the plates were incubated for 30 mins. Then the cells were washed, and the intracellular ATP and numbers of viable cells were determined.

#### Effect of lactic acid on extracellular ATP breakdown rate

Cells were seeded into 6-well plates and pre-incubated for 48 hrs. Then the medium was replaced with a serum-free medium containing CFTR_inh_-172 (20 µM), ATP (1 mM) and either 0 or 10 mM lactic acid. Samples of the extracellular medium were removed after 1, 2 and 10 mins of incubation for analysis of ATP, ADP, AMP and adenosine by HPLC.

#### The role of cAMP-dependent PKA in the activation of CFTR in buffer-perfused rat soleus muscle in vivo and cultured L6 myocytes in vitro

The effect of forskolin on the interstitial ATP of buffer-perfused soleus muscle in vivo was determined: the muscle was perfused with pH 7.4 buffer for a 20 min control period, then forskolin (20 µM) was infused for a further 30 mins, followed by another 10 mins recovery period of buffer-only perfusion. Microdialysis samples were collected for 10 mins each throughout the experimental period for HPLC analysis of ATP.

The effect of forskolin on the extracellular ATP of the cultured myocytes was tested. Cells were seeded into 6-well plates and pre-incubated for 48 hrs. Then the medium was replaced with a serum-free medium: forskolin (100 µM) was added to 3 of the wells on some plates, and lactic acid (10 mM) was added to 3 of the wells on the remaining plates. All plates were incubated for 3 hrs, then the extracellular medium was withdrawn for ATP analysis.

The effect of IBMX on the extracellular ATP of the myocytes, in the absence or presence of lactic acid, was also tested. Cells were prepared as above: 10 mM lactic acid was added to half of the wells on each plate; IBMX (1 mM) was added to all 6 wells on half of the plates, and plates were incubated for 3 hours before collection of the bathing medium for ATP analysis.

The influence of lactic acid and sodium lactate on the intracellular cAMP of the cultured myocytes was examined: cells were seeded into 6-well plates and pre-incubated for 24 hrs before the medium was replaced with serum-free medium. One of the following was then added to each well: no addition, lactic acid (2.5, 5 or 10 mM) sodium lactate (2.5, 5 or 10 mM). All plates were incubated for 30 mins, then the cells were washed and lysed for measurement of cAMP in the lysate. The maximal cAMP response was determined in a separate group of cells by the addition of a range of forskolin concentrations to different wells, followed by incubation for 30 mins, and cAMP determination as above.

The influence of lactic acid on the induction of pCREB was examined in the myocytes. Cells were seeded into 6-well plates and pre-incubated for 24 hrs before the medium was replaced with serum-free medium. Lactic acid (2.5, 5 or 10 mM) was added to some of the wells, and others were left untreated as controls. All plates were incubated for 30 mins, then the cells were washed and lysed for the determination of pCREB and total CREB in the lysates.

The effect of the PKA inhibitor, KT5720, on the extracellular ATP of the cultured myocytes and on the interstitial ATP of the buffer-perfused soleus muscle, in the absence or presence of lactic acid, was tested. Myocytes were seeded into 6-well plates and pre-incubated for 48 hrs. Then the medium was replaced with serum free medium: 10 mM lactic acid was added to half of the wells on each plate; KT5720 (20 µM) was added to all 6 wells on half of the plates, and all plates were incubated for 3 hours before collection of the bathing medium for ATP analysis. The soleus muscle was perfused with pH 7.4 buffer throughout the 90 min equilibration period and for a further 20 min control period. Then lactic acid (5 mM) was infused for 20 mins, followed by a 20 mins recovery period with pH 7.4 buffer perfusion. Infusion of KT5720 (10 µM) was begun, and the lactic acid treatment protocol was repeated in the presence of KT5720. Microdialysis samples were collected for 10 mins each throughout the experimental period for HPLC analysis of ATP.

The effect of lactic acid on PKA activity was determined in the supernatant from the cell lysate: cells were incubated with 0, 2.5, 5 or 10 mM lactic acid for 20 mins, then lysed, and PKA activity was assayed as described above.

#### The influence of lactic acid on the expression of CFTR

L6 myocytes were seeded into 6-well plates and pre-incubated for 24 hrs before the medium was replaced with serum-free medium. In the first series of experiments, lactic acid (2.5, 5 or 10 mM) was added to some of the wells, and others were left untreated as controls. After 30 mins incubation, the cells were washed twice, and the CFTR mRNA expression was determined using RT-PCR. In the second series of experiments, 10 mM lactic acid was added to half of the wells, and the plates were incubated for 3 hrs before the cells were washed and lysed for determination of CFTR protein using Western blot.

#### Involvement of NHE and NCX in the acidosis-induced release of ATP from L6 myocytes

Cells were seeded into 6-well plates and pre-incubated 24 hrs if destined for intracellular cAMP measurements, or 48 hrs if destined for extracellular ATP measurements. Then the medium was replaced with serum free medium: lactic acid (10 mM) was added to 3 of the wells on each plate. The NHE inhibitor, amiloride (100 µM), was added to all wells on some plates, and one of the NCX inhibitors, SN-6 (20 µM) or KB-R7943 (10 µM), was added to all wells of other plates. All plates for cAMP measurements were incubated for 30 mins, then the cells were washed and lysed for measurement of cAMP in the lysate. Plates for extracellular ATP measurement were incubated for either 30 mins (amiloride-treated plates and their controls) or 3 hrs (SN-6- or KB-R7943-treated plates and their controls): then the bathing medium was removed for ATP analysis.

#### Involvement of CFTR in the release of ATP from the contracting skeletal muscle in vivo

Experiments were performed in the free-flow blood-perfused gastrocnemius muscles of anaesthetised rats. Following the 90 min equilibration period, collection of interstitial microdialysis samples was begun. Muscles were given a 20 min rest period, then a 20 min period of 1 Hz contractions, followed by a 20 min recovery period. One of the inhibitors was given as a bolus injection, and the whole procedure was repeated. Inhibitor doses were selected to give plasma concentrations of CFTR_inh_-172 = 20 µM or KT5720 = 10 µM or amiloride = 100 µM or SN-6 = 20 µM. A control series of experiments was performed in which the muscle was subjected to two bouts of contractions without any inhibitor being given, in order to ensure that the release of ATP into the interstitial space would be reproducible in repeated contractions. The ATP concentration in the microdialysate samples was analysed using the bioluminescence assay.

### Statistical analysis

All data are expressed as means ± SEM. Results from the in vivo experiments are expressed as a percentage of their own control for statistical analyses, to minimise the influence of inter-animal variations, and n represents the number of animals used. One-way repeated measures ANOVA followed by the Bonferroni post hoc t-test was performed for comparing multiple treatments within the same group of animals. In the in vitro experiments, n represents the number of tests: Student's t-test was used to compare single groups to their control and one-way ANOVA followed by the Bonferroni post hoc t-test was used for multiple comparisons. In all cases significance was established at P<0.05.

## Results

### Involvement of CFTR in lactic-acid-induced ATP release from skeletal muscle

**Figure 1:**
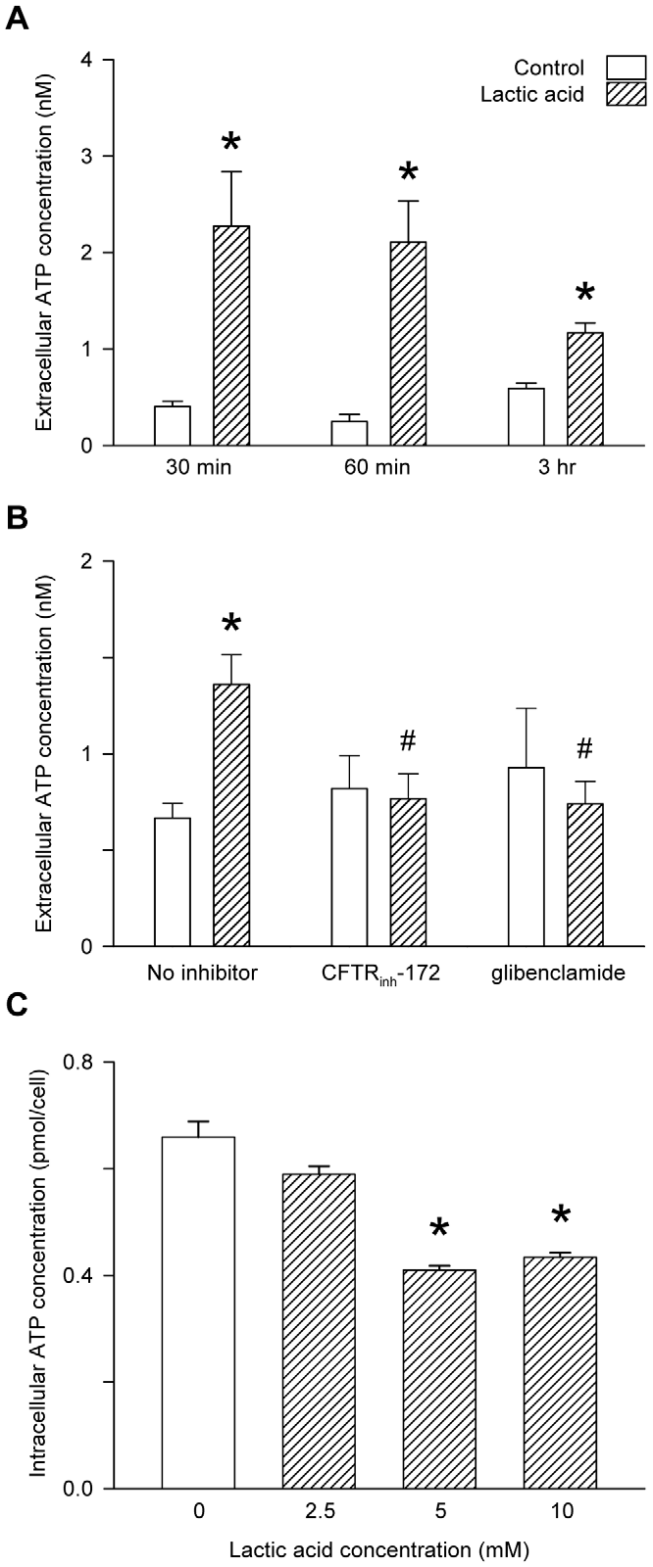
Influence of lactic acid on the extracellular and intracellular ATP of L6 cultured myocytes. A: time-course for the appearance of ATP in the bathing medium during exposure to 10 mM lactic acid. Values are the mean ± SEM of 14–30 values; *, significantly different from its own time-control in Student's t-test. B: appearance of ATP in the bathing medium following 3 hrs exposure to 10 mM lactic acid in the absence of inhibitors or in the presence of CFTR_inh_-172 (20 µM) or glibenclamide (200 µM). Values are the mean ± SEM of 9–12 tests. *, significant difference between the presence and absence of lactic acid in Student's t-test; #, significant difference between the presence or absence of the inhibitor in 1-way ANOVA followed by the Bonferroni post-hoc t-test. C: effect of incubation for 30 mins in different concentrations of lactic acid on the intracellular ATP. Values are the mean ± SEM of 6–12 values; *, significantly different from 0 mM lactic acid in 1-way ANOVA followed by the Bonferroni post-hoc test.

Addition of 10 mM lactic acid to the bathing medium of L6 myocytes reduced the pH from 7.41±0.01 to 6.34±0.06 (n = 11) within the first min; however, the hydrogen ions were rapidly taken up and/or buffered: after 30 mins of incubation, the medium pH had increased to a value not significantly different from the wells without lactic acid, and it remained stable at 7.45±0.02 (n = 10) from 1 to 3 hours of incubation. The extracellular ATP concentration was significantly increased within the first 30 mins of lactic acid treatment; the ATP content of the bathing medium remained elevated for 3 hours (Fig. 1A), but had returned to control after 6 hours. Incubation of the myocytes for 30 mins in a medium containing lactic acid at either 5 or 10 mM significantly decreased the mean cell content of ATP (Fig. 1C). Either the specific inhibitor of CFTR, CFTR_inh_-172, or the CFTR open-pore blocker, glibenclamide, abolished the increase in extracellular ATP induced by 3 hours incubation of the myocytes in a medium containing lactic acid (10 mM) (Fig. 1B).

**Figure 2:**
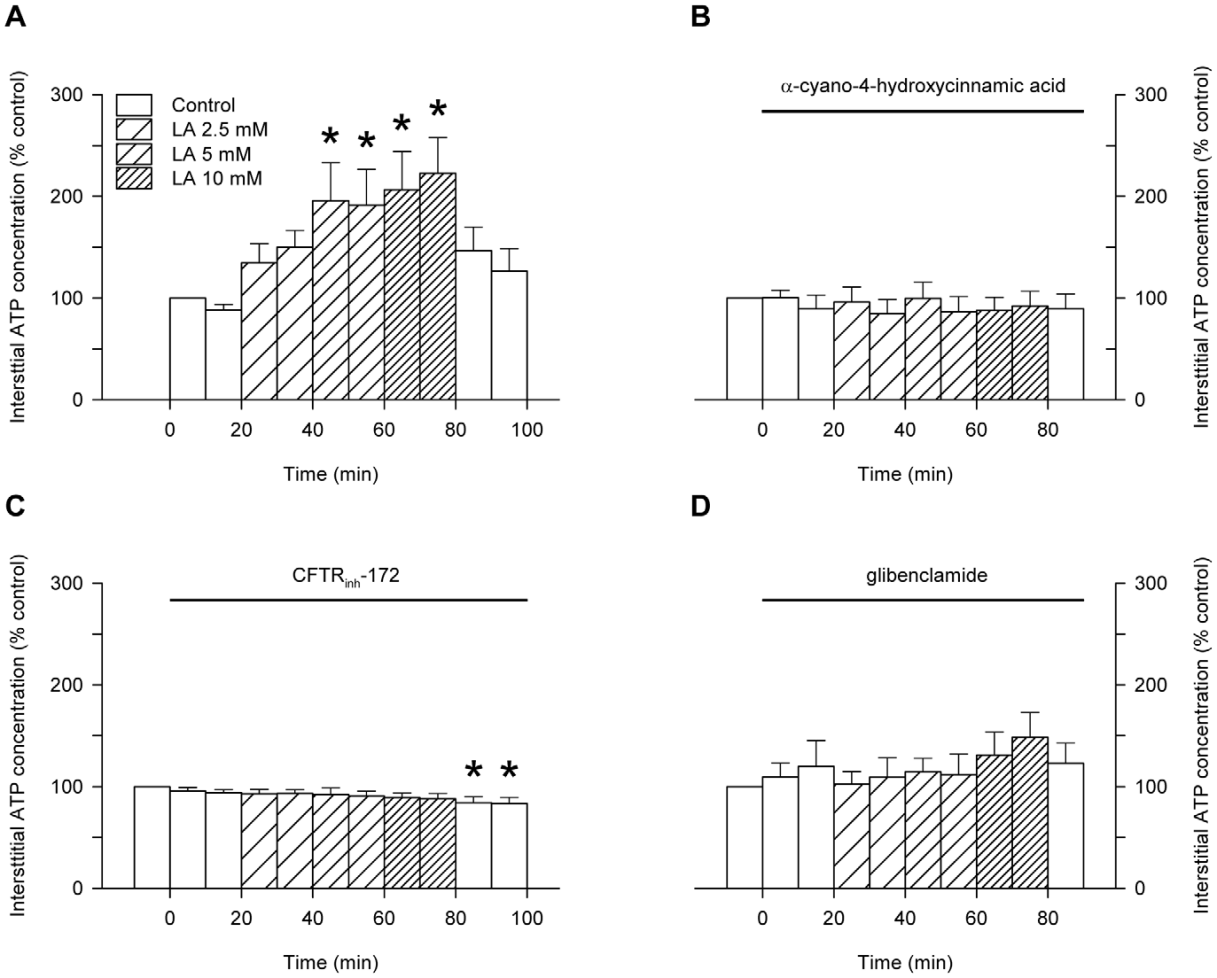
Influence of lactic acid on interstitial ATP of buffer-perfused soleus muscle of anaesthetised rats. Effect of lactic acid on interstitial ATP in the absence of inhibitors (A) or in the presence of α-cyano-4-hydroxycinnamic acid (2 mM; B), CFTR_inh_-172 (20 µM; C) or glibenclamide (200 µM; D). Values are the mean ± SEM of 14–17 values in A, 6 values in B, 7 values in C or 6 values in D. *, significantly different from the pre-control in 1-way repeated measures ANOVA followed by the Bonferroni post-hoc t-test.

The mean interstitial ATP concentration of the rat soleus muscle during perfusion with pH 7.4 buffer was 41±4 nM (n = 36), and the venous pH was 7.22±0.04 (n = 13). Infusion of lactic acid produced dose-dependent increases in the interstitial ATP (Fig. 2A), which were abolished in the presence of the monocarboxylate transporter inhibitor, α-cyano-4-hydroxycinnamic acid (Fig. 2B), the specific inhibitor of CFTR, CFTR_inh_-172 (Fig. 2C), or the CFTR open-pore blocker, glibenclamide (Fig. 2D). None of the inhibitors significantly altered the interstitial ATP during perfusion with pH 7.4 buffer. Sodium lactate, which failed to significantly reduce the venous pH, also failed to significantly increase the interstitial ATP (61±4 nM in control and 72±4 nM with sodium lactate). The interstitial ATP concentration was well-correlated with the decrease in venous pH (r = 0.91).

### Effect of lactic acid on extracellular ATP breakdown rate

There was no significant difference in the rate of extracellular breakdown of ATP between the cells incubated at pH 7.4 or those with 10 mM lactic acid added: over the 10 min incubation period, extracellular ATP decreased to 80.4±4.4% of its starting concentration at pH 7.4, and to 73.6±4.9% in the presence of lactic acid. At the end of the incubation period, the concentrations of both ADP, the product of ecto-ATPase, and adenosine, the product of 5′-nucleotidase, were similar in both groups.

### The role of cAMP-dependent PKA in the activation of CFTR in skeletal muscle

**Figure 3:**
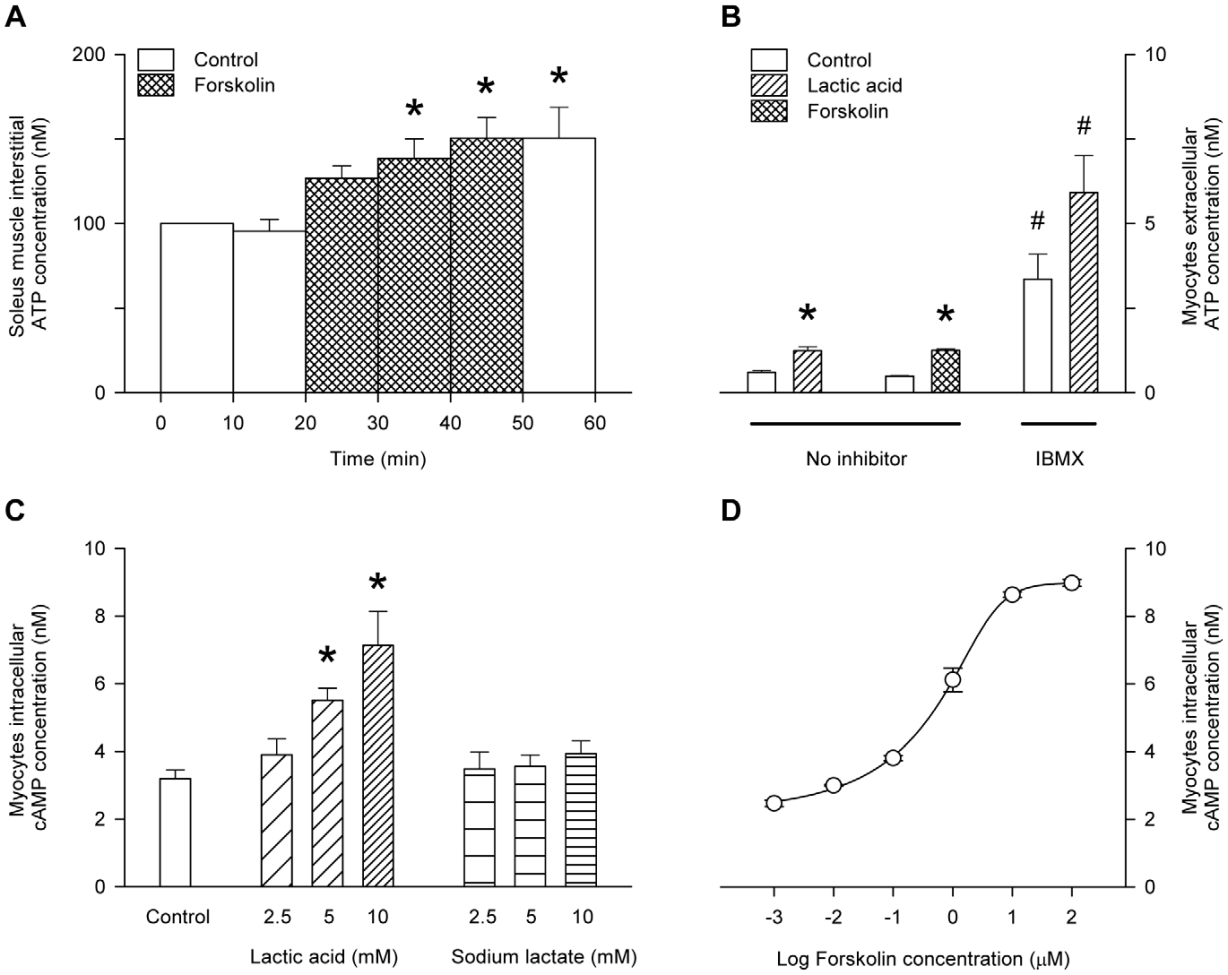
Role of intracellular cAMP in stimulating the increase in extracellular ATP at low pH. A: influence of forskolin (20 µM) on the interstitial ATP of buffer-perfused rat soleus muscle. Values are the mean ± SEM of 5 observations. *, significantly greater than the precontrol in 1-way repeated measures ANOVA followed by the Bonferroni post-hoc t-test. B: comparison of the effect of forskolin (20 µM) or lactic acid (10 mM) in the absence or presence of IBMX on the appearance of ATP in the bathing medium of L6 cultured myocytes. Values are the mean ± SEM of 12–27 tests. *, significantly different from its own control group in Student's t-test; #, significant difference between the absence or presence of IBMX (Student's t-test). C: influence of lactic acid or sodium lactate on the intracellular cAMP of L6 cultured myocytes. Values are the mean ± SEM of 15 values for control and of 6–9 values for treatments. *, significantly different from control in 1-way ANOVA followed by the Bonferroni post-hoc t-test. D: determination of the maximal cAMP response to forskolin in the L6 myocytes: the line shows the 5-parameter sigmoid curve fit (r = 1.0) which predicts a maximal cAMP response of 9.0 nM.

**Figure 4:**
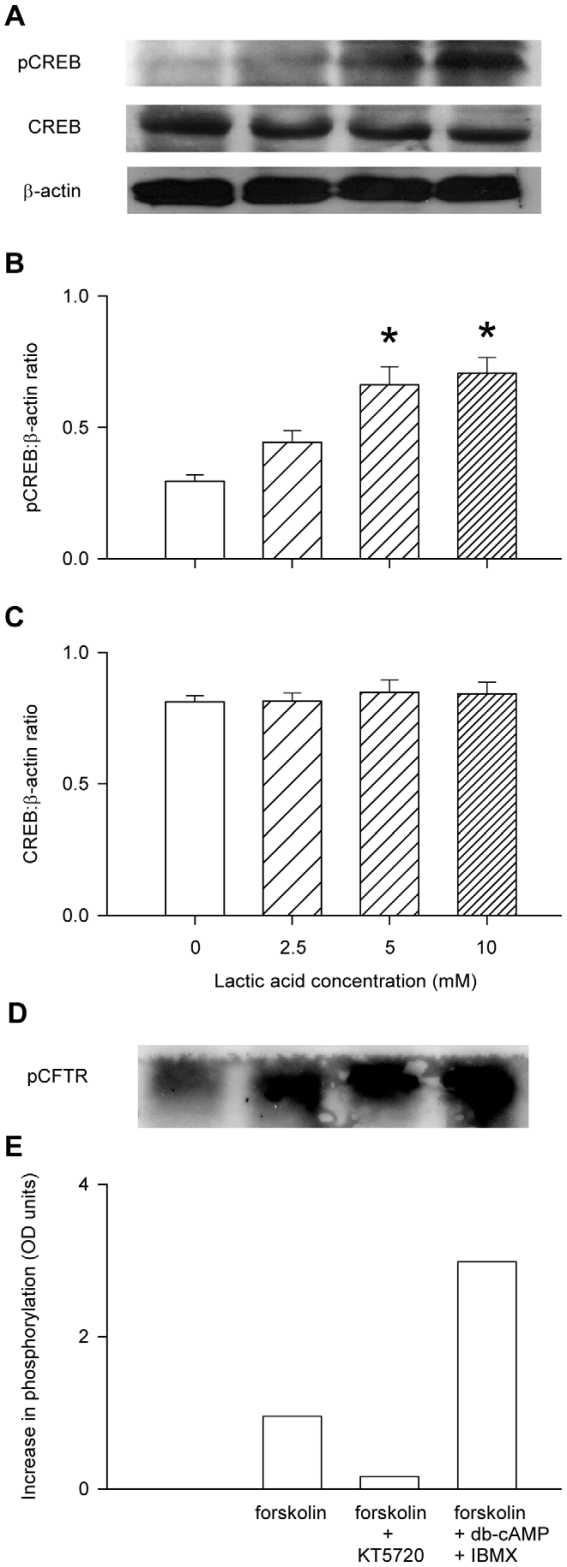
Influence of lactic acid on the induction of pCREB and influence of forskolin on CFTR phosphorylation in L6 cultured myocytes. A: sample gel showing the bands for pCREB, CREB and β-actin, which served as the internal control. Band density for pCREB (B) or total CREB (C) was expressed as a ratio to β-actin. Values are the mean ± SEM of 3 tests. *, significantly different from the value in the absence of lactic acid in 1-way ANOVA followed by the Bonferroni post-hoc t-test. D: sample autoradiograph showing the change in CFTR phosphorylation in response to forskolin (20 µM), forskolin with KT5720, or forskolin plus dibutyryl-cAMP (200 µM) and IBMX (1 mM). E: densitometry analysis of CFTR phosphorylation under the same conditions.

**Figure 5:**
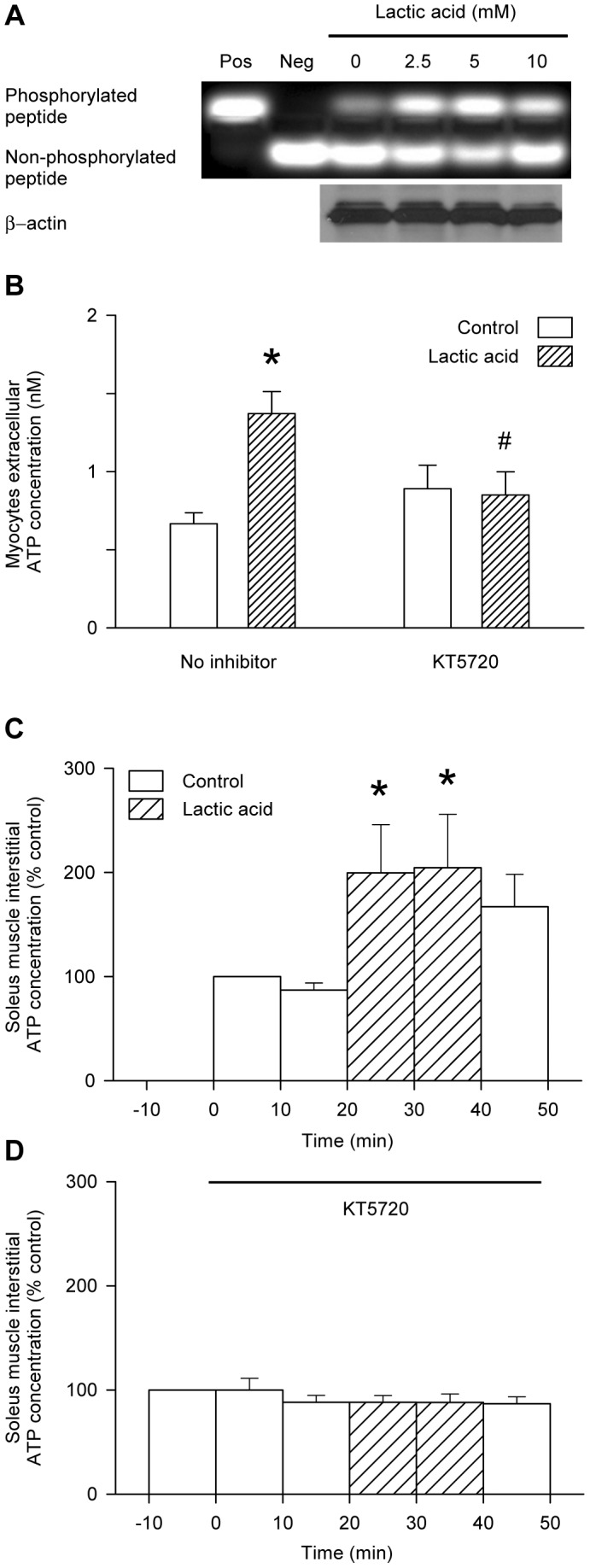
Involvement of PKA in lactic-acid-induced ATP release from cultured myocytes or perfused muscle. A: PKA activity was measured in the supernatant from the L6 myocyte lysate following 30 mins incubation of the cells with the concentrations of lactic acid shown. The upper sample gel shows the separated bands of phosphorylated and non-phosphorylated peptide, and the lower gel shows the internal standard, β-actin, which was measured to confirm that the loading of the gel was kept constant. B: Appearance of ATP in the bathing medium of L6 myocytes exposed to 10 mM lactic acid in the absence or presence of KT5720 for 3 hrs. Values are the mean ± SEM of 9–13 tests. *, significant difference between the absence or presence of lactic acid; #, significant difference between the absence or presence of KT5720 (1-way ANOVA followed by the Bonferroni post-hoc t-test). C & D: Appearance of ATP in the interstitial space of buffer-perfused rat soleus muscle during infusion of lactic acid (5 mM) in the absence (C) or presence (D) of KT5720. Values are the mean ± SEM of 11 (C) or 6 (D) tests. *, significant difference between the absence or presence of lactic acid in 1-way repeated measures ANOVA followed by the Bonferroni post-hoc t-test.

Infusion of forskolin to the buffer-perfused rat soleus muscle in vivo resulted in a significant elevation of the interstitial ATP concentration (Fig. 3A). Similarly, addition of forskolin to the bathing medium of the L6 cultured myocytes produced an increase in extracellular ATP of similar magnitude to that produced by 10 mM lactic acid treatment (Fig. 3B). The phosphodiesterase inhibitor, IBMX, significantly increased the rate of appearance of ATP in the bathing medium of the L6 myocytes, both in the absence and in the presence of lactic acid (Fig. 3B). Treatment of the L6 myocytes with lactic acid produced dose-dependent increases in the intracellular cAMP concentration, whereas sodium lactate failed to elevate intracellular cAMP (Fig. 3C). 10 mM lactic acid raised the intracellular cAMP to 7.1±1.0 nM, whereas the maximal cAMP response to forskolin was 9.0 nM (Fig. 3D). Lactic acid produced dose-dependent increases in the L6 myocyte pCREB content, a surrogate for the activation of PKA by cAMP, whereas total CREB protein remained unchanged (Fig. 4A–C). CFTR phosphorylation was increased by the addition of forskolin alone, and further increased by the addition of forskolin plus dibutyryl-cAMP and IBMX, but the forskolin-induced increase in CFTR phosphorylation was inhibited by KT5720 (Fig. 4D–E). The phosphorylating activity of PKA in the lysate of L6 myocytes was increased following 30 mins incubation in the presence of lactic acid. (Fig. 5A): treatment with 5 mM lactic acid increased the PKA activity from 30.6±4.5 to 37.0±5.4 pmol/min (n = 8; P<0.02, paired t-test), whereas western blot of β-actin (the internal standard) confirmed that total protein loading of the gels remained constant (Fig. 5A). Inhibition of PKA using KT5720 abolished the lactic-acid-induced increases in extracellular ATP of cultured L6 myocytes (Fig. 5B) and interstitial ATP of buffer-perfused rat soleus muscle (Fig. 5C–D).

### The influence of lactic acid on the expression of CFTR

**Figure 6:**
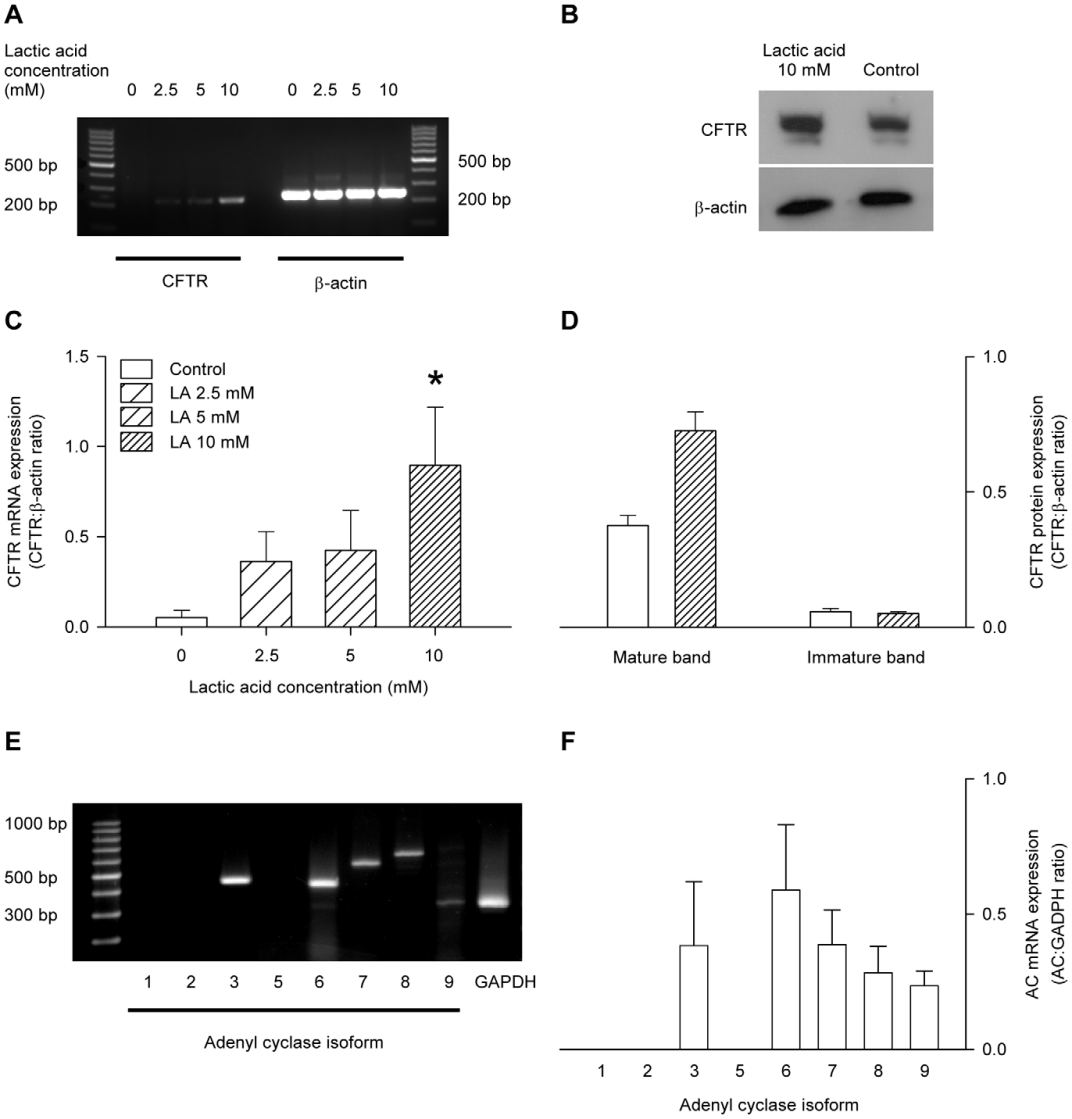
CFTR and AC isoform expression in L6 myocytes. A: sample gel showing RT-PCR of CFTR and the internal standard, β-actin, from cells incubated for 30 mins with the concentrations of lactic acid shown. C. CFTR mRNA expression was normalised as the ratio to β-actin. Values are the mean ± SEM of 4 tests. *, significant difference between the absence or presence of lactic acid in 1-way ANOVA followed by the Bonferroni post-hoc t-test. B: sample gel showing western blot of CFTR protein from cells incubated for 3 hrs in the absence or presence of 10 mM lactic acid. D: CFTR protein expression was normalised as the ratio to β-actin; both the mature (168 kDa) and immature (150 kDa) bands were quantitated. Values are the mean ± SEM of 8–9 tests. *, significant difference between the absence or presence of lactic acid in 1-way ANOVA followed by the Bonferroni post-hoc t-test. E: sample gel showing RT-PCR determination of AC isoforms expressed by L6 myocytes. F: AC mRNA expression was normalised as a ratio to GAPDH. Values are the mean ± SEM of 3 values.

Incubation with lactic acid produced dose-dependent increases in the CFTR mRNA expression, whereas the mRNA for β-actin, the internal standard, remained unchanged (Fig. 6A & 6C). Expression of CFTR protein was also increased by lactic acid treatment: density of the mature fully-glycosylated CFTR band at 168 kDa was significantly increased, whereas, the density of the immature band at 150 kDa was not significantly affected (Fig. 6B & 6D).

### Adenyl Cyclase isoforms expressed by L6 myocytes

Expression of mRNA for the AC isoforms 3, 6, 7, 8 and 9 was detected using RT-PCR (Fig. 6E–F). ACs 1, 2 and 5 were not detected.

### Involvement of NHE and NCX in the acidosis-induced release of ATP from L6 myocytes

**Figure 7:**
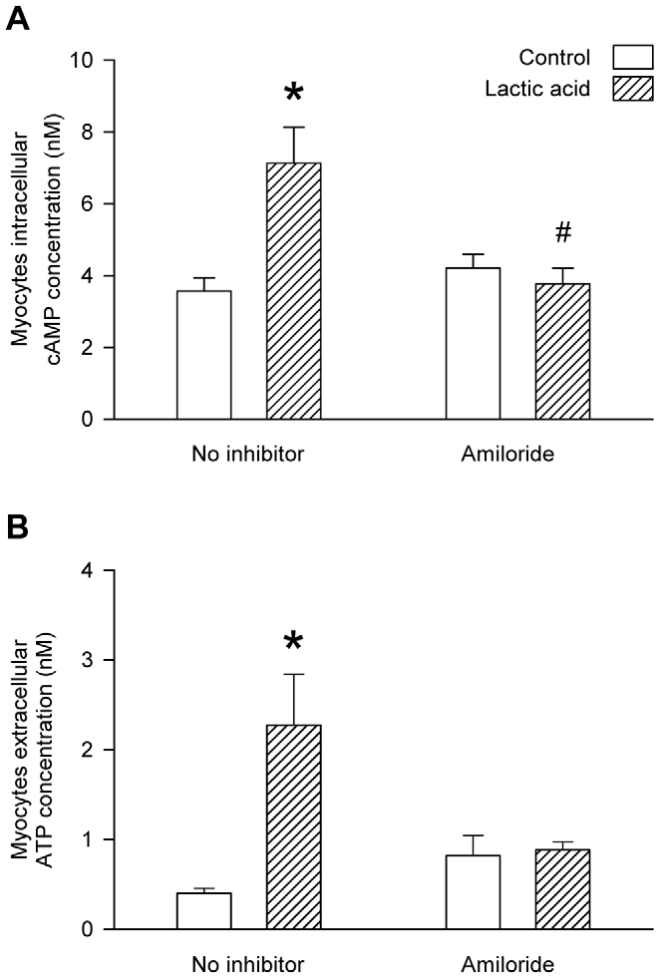
Influence of amiloride on lactic-acid-induced increases in myocytes intracellular cAMP and extracellular ATP. Influence of amiloride on the intracellular cAMP (A) and the extracellular ATP (B) of L6 cultured myocytes incubated for 30 mins in the absence or presence of lactic acid (10 mM). Values are the mean ± SEM of 6–9 tests (A) or 12–27 tests (B). *, significant difference between the absence or presence of lactic acid; #, significant difference between the absence or presence of amiloride (1-way ANOVA followed by the Bonferroni post-hoc t-test).

**Figure 8:**
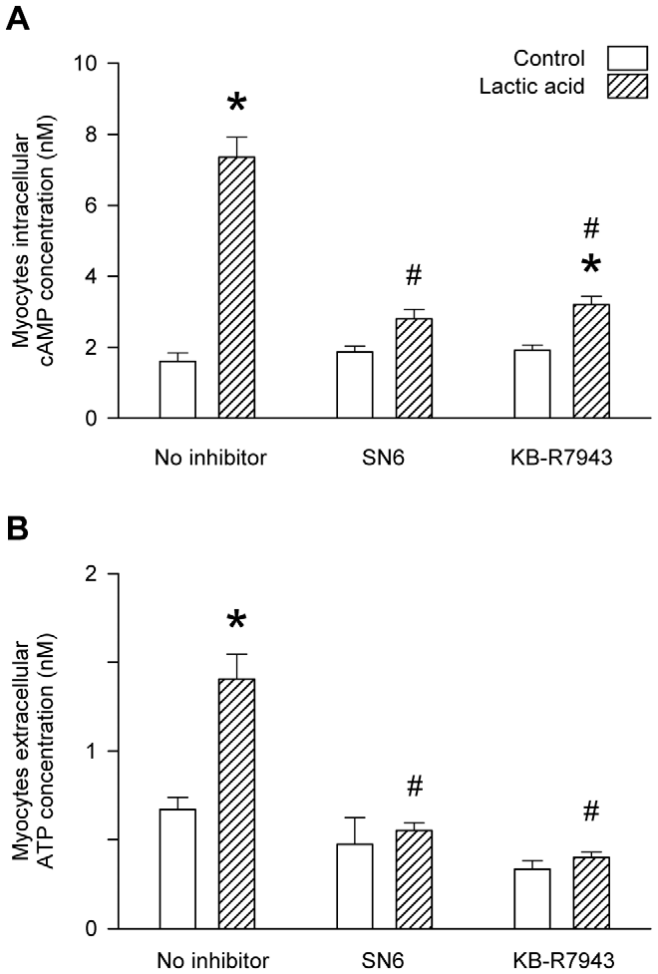
Attenuation of acid-induced increases in intracellular cAMP and extracellular ATP by SN6 or KB-R7943. A: L6 myocytes were incubated in the absence or presence of lactic acid (10 mM) for 30 mins, and values are the mean ± SEM of 6–9 tests. B: myocytes were incubated in the absence or presence of lactic acid (10 mM) for 3 hrs, and values are the mean ± SEM of 12–27 values. *, significant difference between the absence or presence of lactic acid; #, significant difference between the absence or presence of inhibitor (1-way ANOVA followed by the Bonferroni post-hoc t-test).

Treatment with the NHE blocker, amiloride, abolished the lactic-acid-induced increases in intracellular cAMP (Fig. 7A) and extracellular ATP (Fig. 7B) of cultured L6 myocytes. Similarly, the NCX blocker, SN6, abolished the lactic-acid-induced increases in both intracellular cAMP (Fig. 8A) and extracellular ATP (Fig. 8B) of cultured L6 myocytes. The NCX blocker KB-R7943 significantly reduced (but did not fully abolish) the lactic-acid-induced increase in intracellular cAMP (Fig. 8A) but it did abolish the increase in extracellular ATP (Fig. 8B) of the myocytes.

### Involvement of CFTR in the release of ATP from the contracting skeletal muscle in vivo

**Figure 9:**
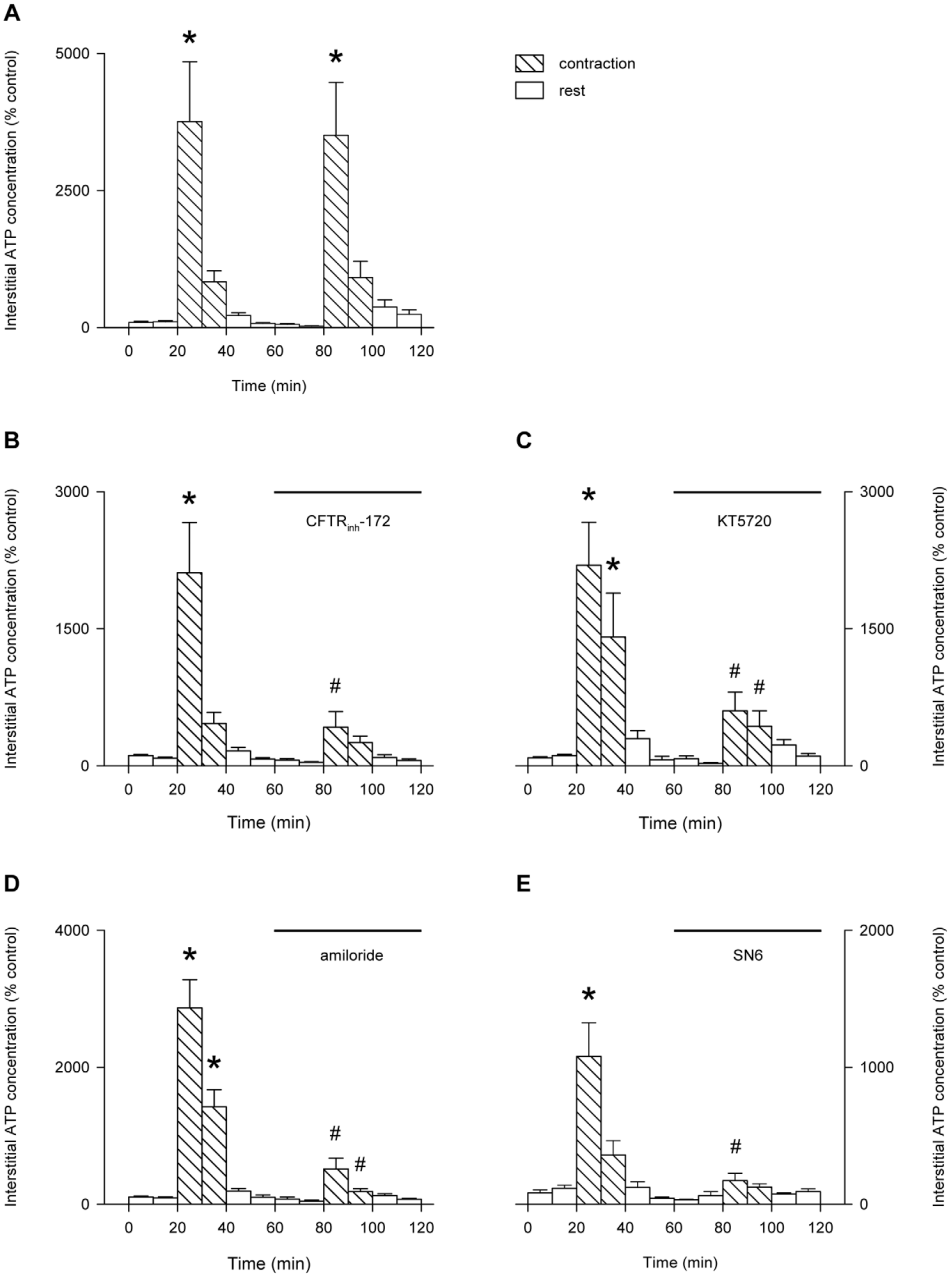
Effect of muscle contractions on interstitial ATP of perfused muscle with or without inhibitors. Blood-perfused rat gastrocnemius muscle was subjected to repeated muscle contractions at 1 Hz in the absence of inhibitors (A) or in the presence of CFTR_inh_-172 (20 µM; B), KT5720 (10 µM; C), amiloride (100 µM; D) or SN-6 (20 µM; E). Values are the mean ± SEM of 6 (A), 11 (B), 7 (C), 8 (D) or 5 (E) tests. *, significant difference between rest and contractions; #, significant difference between absence and presence of inhibitor (1-way repeated measures ANOVA followed by the Bonferroni post-hoc t-test).

Under resting conditions, the interstitial ATP of the blood-perfused rat gastrocnemius muscle was 3.6±0.4 nM (n = 37). Muscle contractions at 1 Hz significantly increased the interstitial ATP to 80.8±11.9 nM in the first 10 mins (P<0.001, 1-way repeated measures ANOVA and Bonferroni post-hoc t-test). Between 10 and 20 mins of contractions, the interstitial ATP was significantly lower than that during the first 10 mins: overall, in the whole group of 37 animals, interstitial ATP remained significantly above control between 10 and 20 mins of contractions, at 33.8±6.0 nM (P<0.001); however, in three of the five subgroups of animals, interstitial ATP was not significantly greater than control in the second contraction sample (Figs. 9A, 9B & 9E), although, in two of these subgroups, it was significantly higher than control in a t-test.

**Figure 10:**
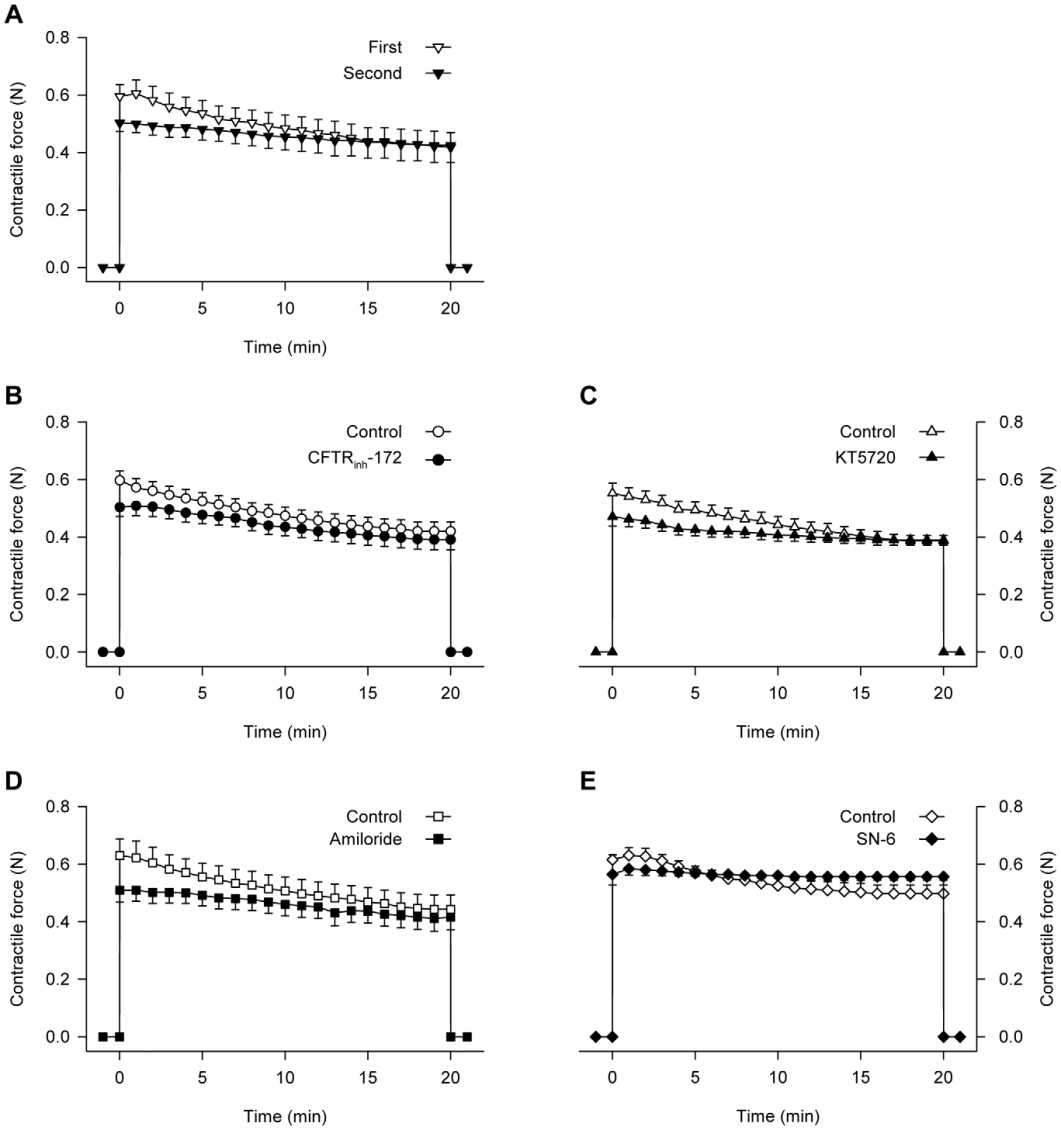
Comparison between force generated in the first and second contractions of perfused muscle. Blood-perfused rat gastrocnemius muscle was subjected to repeated muscle contractions at 1 Hz in the absence of inhibitors (A) or in the presence of CFTR_inh_-172 (20 µM; B), KT5720 (10 µM; C), amiloride (100 µM; D) or SN-6 (20 µM; E). Values are the mean ± SEM of 5–11 tests. There were no significant differences between the first and second contractions in the absence or presence of any inhibitors.

In the control experiment, the increase in interstitial ATP during the second period of contractions was very similar to that during the first contraction (Fig. 9A), indicating that the responses were reproducible in repeated bouts of contractions. However, the contraction-induced increase in interstitial ATP was abolished by the infusion of the specific CFTR inhibitor, CFTR_inh_-172 (Fig. 9B), the PKA inhibitor, KT5720 (Fig. 9C), the NHE inhibitor, amiloride (Fig. 9D) or the NCX inhibitor (Fig. 9E). The force of contraction did not differ significantly between the first and second contraction, either in the absence of drugs (Fig. 10A) or in the presence of any of the inhibitors (Fig. 10B to 10E).

## Discussion

We have proposed a model for the CFTR-regulated release of ATP from skeletal muscle, in which a decrease in intracellular pH stimulates hydrogen ion extrusion from the cell by the NHE, resulting in sodium entry to the muscle, which, in turn, leads to sodium extrusion by the NCX, and allows calcium entry. We hypothesised that the calcium then activates adenyl cyclase, producing a localised increase in cAMP, which activates PKA. PKA phosphorylates the regulatory domain of CFTR, leading to its activation, which results in ATP release from the cell. In this study, we have found strong evidence that cAMP/PKA activation of CFTR is involved in ATP release from muscle; our preliminary experiments using inhibitors of the NHE and NCX are also consistent with the proposed model.

Lactic acid treatment stimulated ATP release from skeletal muscle in vivo and in vitro. The ATP release was stimulated by the lowering of pH rather than the lactate anion, since sodium lactate infusion (which did not significantly reduce pH) failed to stimulate ATP release, the increase in interstitial ATP was negatively correlated with the extracellular pH, and inhibition of the lactic acid uptake abolished the acidosis-induced ATP release. Both specific and non-specific inhibitors of CFTR abolished the lactic-acid-induced ATP release in vivo and in vitro, strongly suggesting that CFTR is involved in the ATP release.

In the present study, lactic acid induced ATP release from the soleus muscle in similar quantities and with a similar time-course to the release from EDL muscle in our previous study [17], suggesting that the release mechanism is similar in both red and white muscle. The similarity between the responses to lactic acid of the perfused muscle in-vivo and the L6 cultured cells in vitro, with or without the blocking drugs, further suggests that the ATP is released from skeletal muscle cells themselves, and that the entire signal transduction mechanism is confined to the skeletal muscle cells alone.

The cAMP/PKA pathway was shown to be involved in the signal transduction pathway for CFTR-regulated ATP release from skeletal muscle: forskolin treatment increased CFTR phosphorylation and stimulated ATP release from muscle both in vivo and in vitro, whilst treatment of the myocytes with lactic acid elevated both the intracellular cAMP and its surrogate, pCREB, and treatment with the phosphodiesterase inhibitor, IBMX [33], enhanced the ATP release from myocytes in the absence or presence of lactic acid. An earlier study on rat soleus muscle also reported that a glycolysis-induced increase in the endogenous production of lactate resulted in the elevation of cAMP [34]; cardiac myocytes from neonatal rats and mice have previously been shown to exhibit cAMP-stimulated CFTR-dependent ATP release [35], [36]. Pretreatment of myocytes with lactic acid increased the PKA activity, whereas inhibition of PKA with KT5720 abolished the lactic-acid-induced release of ATP from muscle both in vivo and in vitro, and inhibited the CFTR phosphorylation induced by forskolin. It has previously been shown that the open-state probability of CFTR in excised membrane patches was increased at low pH, due to an increase in the MgATP affinity of the NBDs [37]: this mechanism may also contribute to the activation of CFTR at low pH in vivo, but cAMP/PKA activation appears to be an essential step in the intact cell, since the acidosis-induced ATP release was completely abolished in the presence of PKA inhibition.

AC exists in 9 transmembrane and at least one soluble isoform [38], which have different regulatory properties. Transmembrane ACs 1, 3 and 8 are stimulated by calcium, through calcium/calmodulin binding [39]; ACs 5 and 6 are inhibited by calcium, and ACs 2, 4 and 7 are not directly affected by calcium [40], although, 2 and 7 are stimulated by protein kinase C (PKC), and may thus be indirectly activated by calcium [41]. AC9 is indirectly inhibited by calcium via calcineurin [40], whereas soluble AC is directly activated by calcium, which increases its affinity for ATP [42]. The calcium/calmodulin-stimulated transmembrane ACs are closely associated with sites of calcium entry to the cell [39], and the calcium signal is thought be localised to a microdomain close to the cell membrane [40].

Our proposed model suggests that calcium entry through the NCX is responsible for activating AC, leading ultimately to CFTR activation and increased ATP release. This mode of entry would be likely to localise the increased calcium concentration to a microdomain near the membrane, which is consistent with our current understanding of AC regulation; logic also dictates that in a tissue such as skeletal muscle, which undergoes elevation of the bulk intracellular calcium concentration during every contraction, a metabolic signal mediated by raised intracellular calcium would need to be compartmentalised to confer sensitivity. Clearly the feasibility of the model is dependent on the expression of a calcium-activated AC isoform in skeletal muscle. We have found mRNA expression of two Ca-activated ACs (AC3 and 8) in muscle, along with two Ca-inhibitable ACs (AC6 and 9), and AC7, which may be indirectly activated by calcium. Further work is required to confirm the protein expression of these ACs, and to determine which of them associate with PKA and/or CFTR.

Incubation of the myocytes with lactic acid increased the CFTR mRNA and protein expression. Phosphorylated CREB is an important regulator of the basal transcription of the CFTR gene, and mediates the effects of PKA on protein synthesis. The cAMP-PKA-activated CREB is able to bind various nuclear proteins and thus to react with several nucleic sequences, particularly the cAMP responsive element (CRE) motif. The CFTR gene contains a CRE in its promoter, at the position of −48 [43]. On the other hand, cAMP-stimulated CFTR gene transcription can also be increased by stimulated expression, which is dependent on nucleotide sequences such as NFκB, SP1, or AP1, known to correspond to inflammation-induced transcription factors [44]. In our experiment, the skeletal myocytes were incubated with lactic acid only for 30 minutes, so the enhancement of CFTR gene expression may be through the stimulated expression mechanism rather than the basal expression mechanism. However, the effects of lactic acidosis on inflammation-induced transcription factors remain controversial, and the mechanism by which lactic acid induced enhancement of CFTR gene and protein expression requires further investigation.

We found that either amiloride (an NHE inhibitor) or SN6 or KB-R7943 (NCX inhibitors) abolished the lactic-acid-induced or contraction-induced ATP release from muscle, suggesting that these exchange proteins may be involved in the signal transduction mechanism leading to ATP release.

In skeletal muscle, NHE mediates the majority of the pH recovery following an acid load [45], by extruding protons from the cell in a 1-to-1 exchange with extracellular sodium ions, which elevates the intracellular sodium [45], [46]. NHE activity is stimulated by a decrease in intracellular pH [46]. Thus, the decreased intracellular pH resulting from lactic acid infusion in our study must have elevated the intracellular sodium. During muscle contractions, it is known that free intracellular sodium is increased [47], which had been attributed to sodium entry during the action potential. However, since the intracellular pH is decreased during muscle contractions [48], sodium entry via the NHE must also contribute to the increased intracellular sodium during muscle contraction.

In cardiac muscle, NHE-mediated accumulation of intracellular sodium drives calcium entry via the NCX [49], [50], and this mechanism is thought to be a major contributor to ischaemic and reperfusion injury [51]. We now propose that a similar mechanism operates in skeletal muscle, and that NHE-mediated extrusion of hydrogen ions during acidosis or muscle contractions also leads to calcium entry via NCX. The finding that amiloride inhibited the contraction- or lactic-acid-induced ATP release from muscle is consistent with the suggestion that NHE-mediated elevation of intracellular sodium is involved in the signal transduction pathway for ATP release, but it remains possible that amiloride directly inhibited the NCX in our study: therefore, further investigation is required to clarify the role of the NHE.

Both NCX1 and NCX3 are expressed in skeletal muscle [52], and exercise training increases the NCX level [53]; the entry of extracellular calcium through the NCX is suggested to play a protective role in high frequency fatigue of skeletal muscle [54]. It has been demonstrated that the NCX can function in reverse-mode in skeletal muscle [55], and that increased cytoplasmic sodium drives reverse-mode operation of the NCX [56], but the possible interaction between NHE and NCX function in skeletal muscle is not well-studied; there is one report that NHE inhibition significantly reduced intracellular calcium accumulation in a muscular dystrophy model [57].

Our data suggest that CFTR-regulated release makes a major contribution to the increased interstitial ATP of contracting muscle in vivo, that PKA is involved in the activation of CFTR during the muscle contractions, and that both the NHE and NCX may be involved in initiating the signal transduction pathway. None of the inhibitors significantly reduced the contractile force of the muscle, and therefore, the attenuation of ATP release in their presence could not be explained by any diminution in the work of the muscle; furthermore, in the absence of inhibitors, the release of ATP to the interstitial space remained extremely reproducible in repeated contractions, which rules out any time-dependent decline in muscle function.

We did not use inhibitors of ATP breakdown or adenosine uptake in this study: thus the extracellular and interstitial ATP concentrations represent the resultant of the processes of release, uptake and breakdown. This has the advantages that it more closely represents the situation in vivo and avoids any possible side-effects from inhibitors, but it means that an increased extracellular concentration is only an indirect measure of increased ATP release. However, we are confident that an increased extracellular ATP concentration does reflect an increase in ATP release, firstly because the increased extracellular ATP was accompanied by a decrease in intracellular ATP, secondly because both ATP breakdown and adenosine uptake are concentration-driven processes, whose rates would increase in proportion to the extracellular ATP, and thirdly because we have confirmed that the rate of extracellular ATP breakdown was unchanged at low pH.

The inter-animal variation in the interstitial ATP level was unlikely to result from tissue damage during probe insertion, firstly because the probe was perfused for 1½ hours before samples were collected, during which time the interstitial ATP decreased to a steady level, and secondly because those animals having lower interstitial ATP under control conditions, also tended to have lower levels during acid infusion or muscle contractions. It is more likely that the variation arose either from a genuine inter-animal variability in the capacity for ATP release, or else from differences in the probe recovery between different experiments. In order to minimise differences in probe recovery, we always used a brand new probe for each experiment, and the recovery measured in the in-vivo control experiments appeared to be quite consistent. We were unable to use an internal standard to measure recovery for individual samples, as the entire sample collected was required for the ATP analysis. Therefore, we normalised the in-vivo concentrations to a percentage of their own control, which would effectively correct for any variability in either probe recovery or capacity for ATP release.

After correction for the probe recovery, the control interstitial ATP was in the range 20–170 nM in the buffer-perfused muscle, and 2–20 nM in the blood-perfused muscle, which is in good agreement with our own previous measurements [11], [17], and values determined by other groups for muscle [2], [15], [58] and other tissues [59], [60]. Interstitial ATP increased 1000–3000% in the first 10 mins of muscle contractions, which is similar to the increase during leg extensor exercise in humans [2], [15]. It decreased between 10 and 20 mins of contractions, remaining significantly above control in only half of the muscles. This could result from decreased ATP release from the muscle cells or increased removal of ATP from the interstitial space. The contractile force was well-maintained throughout the contractions, which rules out contraction failure as a cause for decreased ATP release. However, in prolonged sub-maximal contraction of rat muscle, the intracellular pH is reported to reach its minimum value after 5–10 mins, and to increase back to a value only slightly lower than rest if the exercise is continued to the steady state [61]–[63]: if the decreased intracellular pH is one of the signal transduction steps leading to ATP release, then it is possible that the rebound in pH might account for a reduction in ATP release between 10 and 20 mins of contractions.

In conclusion, we have shown that CFTR is involved in the ATP release from skeletal muscle during acidosis, both in vivo and in vitro, and that CFTR-regulated ATP release makes a major contribution to the ATP release from contracting skeletal muscle. CFTR was activated mainly through the cAMP/PKA pathway, and our data are consistent with a mechanism whereby both the NHE and the NCX are involved in the signal transduction mechanism relating the reduction in intracellular pH to the activation of CFTR.
