# Correction: cAMP/Protein Kinase A Activates Cystic Fibrosis Transmembrane Conductance Regulator for ATP Release from Rat Skeletal Muscle during Low pH or Contractions

**DOI:** 10.1371/annotation/bca273e5-7448-4773-a4e4-fe538be554da

**Published:** 2013-08-21

**Authors:** Jie Tu, Lin Lu, Weisong Cai, Heather J. Ballard

A funding organization and grant were incorrectly omitted from the Funding Statement. The Funding Statement should read: "This work was supported by the Hong Kong Research Grants Council Direct Allocation grant numbers 10207994-14464-21400-323-01, 10400678-14464-21400-323-01 and 10401287-14464-21400-323-01, the Li Ka Shing Foundation, grant number 2046-14464-21400-N01-01, the University of Hong Kong, grant number 21372567-14464-21400-N01-01, and the National Natural Science Foundation of China grant number 30900594. The funders had no role in study design, data collection and analysis, decision to publish, or preparation of the manuscript." 

